# Risk Perception and Psychosocial Factors Influencing Exposure to Antimicrobial Resistance through Environmental Pathways in Malawi

**DOI:** 10.4269/ajtmh.24-0253

**Published:** 2024-12-03

**Authors:** Kondwani Chidziwisano, Derek Cocker, Taonga Mwapasa Kumwenda, Steve Amos, Nicholas Feasey, Tracy Morse

**Affiliations:** ^1^ Centre for Water, Sanitation, Health and Appropriate Technology Development, Malawi University of Business and Applied Sciences, Chichiri, Malawi;; ^2^ Department of Environmental Health, Malawi University of Business and Applied Sciences, Chichiri, Malawi;; ^3^ Department of Civil and Environmental Engineering, University of Strathclyde, Glasgow, United Kingdom;; ^4^ Malawi-Liverpool Wellcome Programme, Kamuzu University of Health Sciences, Blantyre, Malawi;; ^5^ Department of Clinical Sciences, Liverpool School of Tropical Medicine, Liverpool, United Kingdom

## Abstract

Antimicrobial-resistant (AMR) bacteria are prevalent in household and environmental settings in low-income locations. However, there are limited data on individuals’ understanding of AMR bacteria exposure risks in these settings. A cross-sectional study was conducted to identify individual risk perception of AMR bacteria and its associated behavioral determinants at the household level in urban, peri-urban, and rural Malawi. We conducted interviews with 529 participants from 300 households (*n* = 100 households/site). The risk, attitude, norms, ability, and self-regulation model was used to assess psychosocial factors underlying AMR bacteria exposure through animal feces, river water, and drain water. Analysis of variance was used to assess the difference between doers and non-doers of the three targeted behaviors: use and contact with river water, contact with drain water, and contact with animal feces. There was limited understanding regarding human–environmental interactions facilitating AMR bacteria transmission across all sites, and as such, the perceived risk of contracting AMR infection was low (41%; *P* = 0.189). Human contact with animal feces was seen as risky (64%) compared with contact with river and drain water (17%). Urban participants perceived that they were at greater risk of AMR bacteria exposure than their rural counterparts (*P* = 0.001). The perception of social norms was favorable for the targeted behaviors (*P* = 0.001), as well as self-reported attitude and ability estimates (self-efficacy; *P* = 0.023), thus indicating the role of psychosocial factors influencing the human–environment interaction in AMR bacteria transmission. Our findings underscore the need for combined infrastructural improvements and behavior-centered AMR bacteria education to drive behavioral changes, benefiting both AMR infection mitigation and broader One Health initiatives.

## INTRODUCTION

Antimicrobial resistance (AMR) constitutes one of the most serious global public health threats of the twenty-first century.^1^^,^^2^ Globally, an estimated 4.95 million people die annually because medicines have become ineffective against diseases that were previously treatable. This accounts for 27.3 deaths per 100,000 in sub-Saharan Africa.^3^ With increasing evidence that environmental factors are accelerating the proliferation and spread of AMR bacteria,^4^ particularly in low- and middle-income settings,^5^ there has been global recognition^6^ that a whole-system One Health Approach is needed to address AMR infection.

Recent studies across low and middle income countries (LMICs) have identified environmental pathways by which humans may be exposed to AMR bacteria,^7^^–^^9^ all of which highlight the invaluable role environmental health interventions can play in the prevention and control of AMR bacteria transmission among humans, animals, and the environment.

Evidence from these studies has revealed a link among the environment, animals, and human exposure to AMR bacteria. Most recently, studies conducted in Malawi have confirmed a high prevalence of AMR bacteria in surface water and human and animal feces.^7^^,^^8^ All these routes are commonly associated with the transmission of infectious diseases.^10^ It is widely understood that infrastructure alone cannot resolve these complex One Health challenges and that we therefore need to understand the determinants that drive human behavior and risk in these settings to ensure interventions are context-appropriate, effective, and, when possible, sustainable. However, there has been no reported research undertaken to understand how people living within these high-risk environments perceive their risk of exposure to AMR bacteria from their day-to-day interactions, including behaviors and behavioral determinants that bring people into contact with high-risk areas of the environment. If we are to develop effective interventions that not only address AMR bacteria exposure but also provide other social benefits, then we need to understand whether the behavioral determinants associated with AMR bacteria exposure and their pathways may differ from those of standard environmental health interventions.

With this in mind, we conducted a cross-sectional study using the risks, attitudes, norms, abilities, and self-regulation (RANAS) model^11^ to explore the perception of risk to AMR bacterial infections from environmental pathways (drain water, river water, and animal feces) and to establish the behavioral (i.e., psychosocial) factors that bring people into contact with the environment (i.e., drain water, river water, and animal feces). The RANAS model was used because it presents “five block” psychosocial factors (i.e., risk factors, attitude factors, norm factors, ability factors, and self-regulation factors) that should be applied to understand the psychosocial factors of a particular population to determine a specific behavior. Previously, the RANAS model has been successfully applied to understand behavioral determinants related to water treatment, sanitation, food hygiene, and hand-washing behaviors.^12^^–^^14^ This research was site-specific and was conducted in rural, peri-urban, and urban locations in Malawi to understand the variation across these populations.

## MATERIALS AND METHODS

### Study area.

This research was part of a large study previously described elsewhere,^15^ which selected study sites representing urban, peri-urban, and rural settings in Malawi to enable the evaluation of variations in One Health factors, including water, sanitation, and hygiene (WASH) behaviors. Thus, three study locations were identified: 1) Ndirande in the Blantyre District, representing the urban setting, 2) Chileka in the Blantyre District, representing the peri-urban setting, and 3) Traditional Authority Kasisi in the Chikwawa District, representing the rural setting. Ndirande is a large urban settlement with high-density housing 4 km from the geographical center of Blantyre, the second city of Malawi, where 15% (109,164) of the Blantyre population resides.^16^^,^^17^ Ndirande has various surface water streams, including the Nasolo River, which runs across the township. Because not all residents are connected to the municipal water system, the surface water bodies in Ndirande are used for various purposes, including washing clothes and other household items. In addition, the streams serve as a playing area for children and a waste dumping site for the majority of households because there is improper waste management.^18^ Ndirande has no proper sewerage system, and latrines constructed very close to houses are mainly used for fecal defecation. Whenever possible, pit latrines are constructed on the edges of rivers to allow for the easy disposal of their contents into the rivers. Stagnant water and sewage pools are a common sight in Ndirande, especially in the rainy season (i.e., November to April). Being an urban location, the rearing of animals is on a smaller scale compared with the peri-urban and rural study locations.^7^ Chileka, which is situated ∼15 km from the center of Blantyre, is a peri-urban administrative ward on the northern outskirts of Blantyre City, where access to safe water is mostly through piped water supply and boreholes. However, shallow wells are also present, which are mostly used for watering crops and other domestic purposes, including washing clothes and kitchen utensils, to a lesser extent. The rural Chikwawa District has a population of ∼450,000 and is situated in the southern Shire Valley, and its border is 50 km from Blantyre.^19^ Subsistence farming and the rearing of animals is common,^7^ and given its low-lying situation, it is historically prone to flooding.^20^ Access to improved sanitation in the study locations is mostly through pit latrines (i.e., 37.3% in Blantyre and 42.4% in Chikwawa).^21^

### Study population and sampling.

In total, 600 study participants were to be recruited from 300 participating households (i.e., 100 households from each study location). The household selection criteria of the 300 households have been reported elsewhere.^15^ In each household, a maximum of two participants were recruited: 1) female or male household members responsible for household chores, such as preparing food, washing clothes, and taking care of children, and 2) head of households.

### Data collection.

Data collection was conducted from October to November 2020. A RANAS model-based questionnaire was used to collect data from all the study participants to identify the psychosocial factors associated with individual perceptions of AMR infection risk via environmental pathways.^11^ The RANAS model has been developed using psychological theories.^22^^–^^25^ Specifically, the questionnaire, administered in the local language (Chichewa) included closed questions about demographics, access to safe water, and sanitation facilities. Regarding AMR bacteria awareness, the study participants were asked to describe what they understood about AMR bacteria, AMR infection risk perception, psychosocial factors about AMR bacteria exposure through drain water and river water, and animal feces management (Table 1). Five-point rating scale questions (ranging from “not at all” to “very much” scale) were used to capture data on specific RANAS variables about drain water, river water, and animal feces. Five experienced, well-trained research assistants who were fluent in the local language (Chichewa) collected the data in all three study locations. The questionnaire was pretested before data collection, which helped to eliminate irrelevant questions, and further alteration was performed on the key questions to improve understanding.

**Table 1:** Questions on targeted behaviors

Behaviors	Answer Format
Do you use river water at this household for domestic purposes (e.g., washing utensils, watering vegetables, washing laundry, bathing, etc.)?	Not at all to very much (five-point rating scale)
Do you come in contact with drain water (e.g., when crossing a drain, using drain water for something like washing or construction)?	Not at all to very much (five-point rating scale)
Do you directly or indirectly come into contact with animal feces? Give an example of being in contact with animal feces indirectly (e.g., through water or utensils).	Not at all to very much (five-point rating scale)

## STATISTICAL ANALYSES

KOBO collect^26^ (Kobo Inc. MA, https://www.kobotoolbox.org/about-us/) on Android tablets (Samsung Electronics, Ridgefield Park, NJ) was used to collect the data, which were later exported to Microsoft Excel (Microsoft Corporation, Redmond, WA) and quality checked before being exported to Statistical Package for Social Sciences (SPSS, IBM, Armonk, NY), where a frequency distribution of the demographic characteristics using descriptive statistics was plotted. In IBM SPSS version 25, the PROCESS macro for SPSS was used to undertake all statistical tests (IBM). The household RANAS model-based data were analyzed using an analysis of variance (ANOVA) mean comparison analysis to determine the differences between the doer and non-doer contextual and psychosocial factors for the targeted behaviors.

To measure the three targeted behaviors (i.e., contact with drain water, use of river water, and contact with animal feces), the study participants were asked how often they were in contact with drain water, how often they used river water, and how often they were in contact with animal feces. Frequencies were measured on a five-point scale. All factors falling at or below the mid-three-point value on a scale of one to five were considered non-doers of the targeted behaviors, whereas those factors greater than or equal to four were doers of the behavior, and the mean score for each targeted behavior was calculated. Contact with drain water, the use of river water, and contact with animal feces were dependent variables, whereas behavioral factors of the RANAS model were independent variables. A single item was used to measure perceived vulnerability of antimicrobial resistance and other psychosocial factors were measured with multiple items. For each targeted behavior, the significant factors among those noted with ANOVA calculation were further analyzed (i.e., any factor at *P* <0.05 using ANOVA) with effect size, *d*, where Cohen’s *d* values were as follows: small: ≤0.20, medium: ≤0.50, and large: ≥0.80.

## RESULTS

### Sociodemographic characteristics.

**Figure 1.**
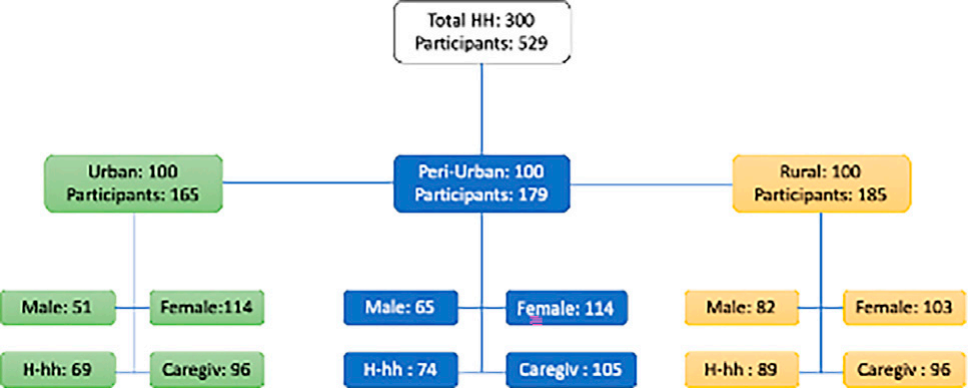
Distribution of study participants according to location and sex. HH = household; H-hh = head of household; Caregiv = caregiver.

The 529 study participants from 300 recruited households were composed of 63% females and 37% males, with an average age of 26 years (Figure 1). It was not possible to collect data from a male and a female participant in every household because of logistical challenges; men were reported as being away from home to perform various activities for income generation, particularly in the urban areas (Figure 1). The mean number of residents per household across the study was 4.5, with the urban, peri-urban, and rural sites having 4.6, 4.6, and 4.2 members per household, respectively, illustrating limited variations in household density between the regions. Household income was mainly from subsistence farming and small-scale business activities in all the study locations, with the majority (78%) of the households earning less than $1.90 per day.

The primary source for drinking water in households was from communal distribution points, including boreholes (48.7%; *n* = 153) and water kiosks (25.2%; *n* = 79), or piped water, either inside (7.6%; *n* = 24) or outside (16.9%; *n* = 53) the household compound. There were regional differences in the primary water source used, with boreholes frequented by rural (84.3%; *n* = 86) or peri-urban households (56.1%; *n* = 60) and municipal kiosks used by urban households (61.0%; *n* = 64). The use of unprotected wells (0.6%; *n* = 2) or surface water, such as rivers or ponds (0.3%; *n* = 1), for drinking was reported in a low number of households. However, households indicated that unprotected water sources (from the river and drains inclusive) were used for other domestic purposes (urban: 9%; peri-urban: 16%; and rural: 3%) such as washing clothes and household utensils. Importantly, it was noted that water from the river and drains (mostly in the rainy season) was used as an alternative when one could not access the safe water from boreholes or taps because of the non-functionality of the water points. It was further reported that children play with drain water mostly during the rainy season.

In terms of sanitation, the study locations had an overall toilet coverage of 86%. However, the rural location had the lowest toilet coverage (72%) compared with urban (93%) and peri-urban (95%) locations. The findings align with high open defecation levels observed in rural (15%) compared with urban (7%) and peri-urban (5%) locations. Forty-two percent of households reported sharing toileting facilities with other households within the compound or wider community. Given the nature of high-density housing, this practice was more often identified at the urban site (62.1%). The study also established that toilet facilities in urban locations were generally unhygienic, and slightly over half of them were almost full. However, the peri-urban and rural study areas mainly featured traditional unimproved toilets that were prone to collapse during the rainy season. In terms of drainage systems, all study areas had either no or poorly constructed drainage infrastructure, which resulted in uneven distribution and accumulation of surface water mostly in the rainy season.

In total, animal feces was observed in 57% of the households, with those in rural (83%) and peri-urban (56%) settings having the highest environmental animal fecal contamination, compared with urban settings (31%). This tallied with the presence of domestic or livestock animals observed in and around the households, with rural (63%) and peri-urban (45%) households having animals observed in the household complex more often than those in the urban setting (32%). Correspondingly, it was reported that 58.7% of households cohabitate with domestic or livestock animals, with 36% (*n* = 36), 59% (*n* = 59), and 81% (*n* = 81) of households in the urban, peri-urban, and rural sites owning ≥1 animal.

### Knowledge and AMR infection risk perception.

**Figure 2.**
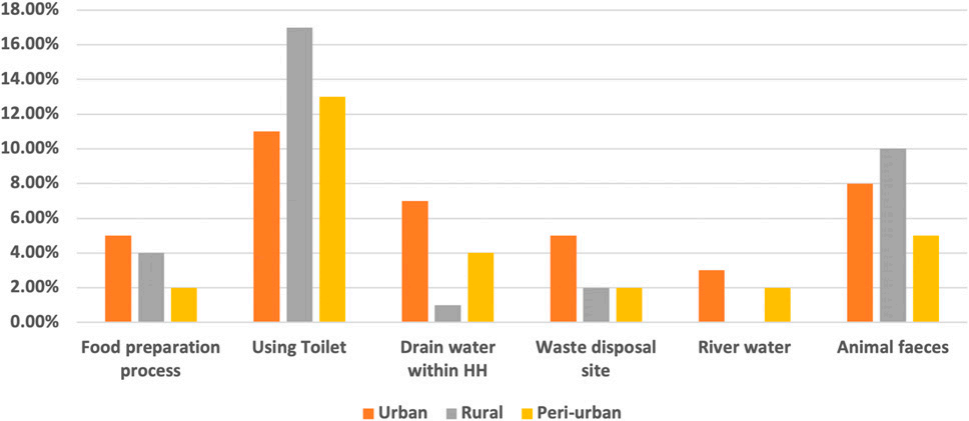
Perceived potential pathways for antimicrobial-resistant infection environmental transmission.

Fifty-three percent of the study participants from the three study locations were aware of AMR bacteria, and the level of knowledge did not differ significantly among the three study locations (*P* = 0.063) and study participants (male versus female: *P* = 0.061). Most of the study participants (76%) who demonstrated knowledge about AMR bacteria were aware of individuals who had been diagnosed with an AMR bacteria-related infection. With this in mind, the study established that fewer than one-third of participants (29%) who had demonstrated knowledge about AMR infection perceived themselves to be at risk of an AMR bacteria-related infection. There was a slightly higher perception of risk in urban compared with rural areas (i.e., urban = 44%, peri-urban = 36%, and rural = 20%). Female study participants felt that they were at greater risk of AMR infection compared with their male counterparts (*P* = 0.001). Most respondents (64%) associated the risk of AMR infection transmission with direct exposure to human and animal feces. Very few respondents (17%) identified indirect exposure through drain and river water as areas of risk in the transmission of AMR bacteria at the household level (Figure 2). However, the study participants expressed limited knowledge of how being in contact with or using drain and river water facilitated the transmission of AMR bacteria.

**Figure 3.**
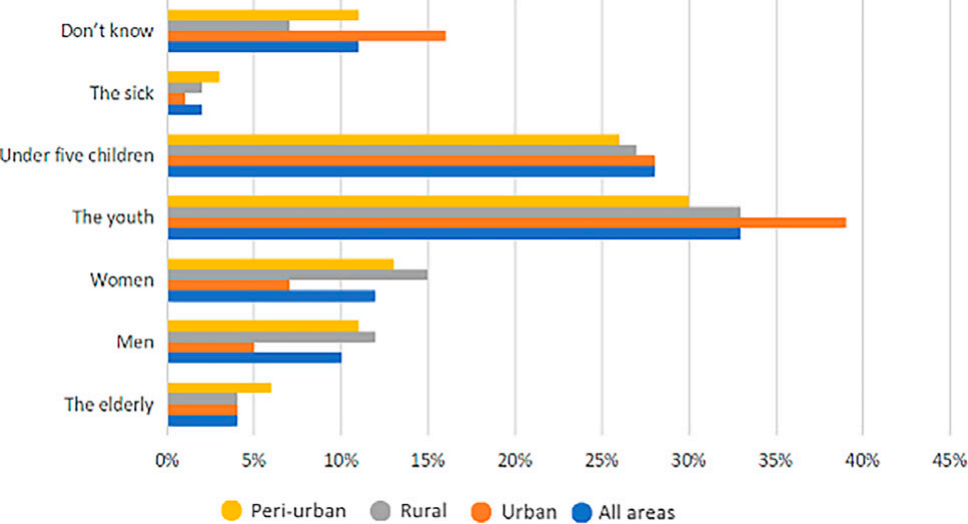
Groups perceived to be at risk of antimicrobial-resistant infection.

When asked whom they perceive to be at high risk of AMR infection transmission, the study participants in all study locations referred to the youth (mainly in the adolescent age range) and children younger than five years as the most vulnerable groups (Figure 3). The participants perceived that the young children and youth’s immunity is still under development; hence, they believe that children and youth are exposed to AMR infections.

### Psychosocial factors.

Risk, attitude, norms, abilities, and self-regulation model-based questions were asked to understand psychosocial factors that contributed to study participants being exposed to AMR bacteria through the use of river water, contact with drain water, and contact with animal feces.

#### Use of river water for domestic purposes.

On the perceived risk of being exposed to AMR bacteria through the use of river water for domestic purposes, the “doers” were those participants who perceived no risk of AMR infection when they used river water for household activities (i.e., bathing, washing clothes, and kitchen utensils). In contrast, the “non-doers” were those who perceived a risk of being exposed to AMR bacteria when they used river water for household activities (i.e., bathing, washing clothes, and kitchen utensils). Overall, the study found that all five behavioral factors were important when considering the use of river water at the household level as a risk to AMR bacteria exposure; norm factors (i.e., others’ behaviors [relative], others’ behaviors [neighbor], others’ approval, personal obligation, ability factors [i.e., confidence in performance, confidence in performance (hurry)], and self-regulation factors [i.e., attention, importance, and commitment]) significantly differed between the “doers” and “non-doers” in all three study settings (Table 2). This implies that the “doers” perceived that their relatives and neighbors used river water for domestic purposes because they did not relate it with AMR infection transmission; those in authority did not perceive any risk of being exposed to AMR bacteria when using river water, and as such, they did not discourage others from using river water for domestic purposes. Relatedly, the “doers” showed more confidence in using river water whenever they were in a hurry and paid less attention and commitment to avoiding using river water than the “non-doers.” The time-consuming factor was found to be significant in rural (*d* = 1.1) and peri-urban (*d* = 1.24) settings, where the “doers” in rural and peri-urban locations felt that it was less time-consuming for them to fetch water from the river for domestic purposes. However, disgust was a significant factor for non-doers in avoiding the use of river water for domestic purposes in peri-urban (*d* = 0.73) and urban (*d* = 0.65) settings. When the psychosocial factors were compared between male and female study participants, there was no significant difference between the two groups in almost all behavioral factors of interest (Supplemental Tables 1 and 2).

**Table 2:** Use of river water: Doer and non-doer risk, attitude, norms, abilities, and self-regulation psychosocial factor means compared with analysis of variance

Factor Group	Behavioral Factors	Contact with River Water								
		Rural Setting			Peri-Urban Setting			Urban Setting		
		Cohen’s *d*	Doer’s, *M* (SD)	Non-Doer’s, *M* (SD)	Cohen’s *d*	Doer’s, *M* (SD)	Non-Doer’s, *M* (SD)	Cohen’s *d*	Doer’s, *M* (SD)	Non-Doer’s, *M* (SD)
Risk factors	Vulnerability	0.56*	4.75 (0.62)	4.63 (0.81)	0.7*	4.27 (1.19)	4.64 (0.82)	0.98*	3.88 (1.25)	4.82 (0.56)
Attitude factors	Pleasant	0.92*	2.83 (1.403)	1.67 (1.105)	0.90*	2.96 (1.71)	1.66 (1.11)	0.90*	3.00 (2.14)	1.49 (1.04)
	Time-consuming	1.1*	1.75 (1.05)	3.35 (1.76)	1.24*	1.62 (1.17)	3.39 (1.65)	n.s.	3.75 (1.49)	3.52 (1.39)
	Disgusted	n.s.	2.75 (1.658)	3.42 (1.703)	0.73*	2.08 (1.23)	3.19 (1.78)	0.65*	2.88 (1.64)	3.91 (1.54)
	Care	n.s.	3.25 (1.815)	2.84 (1.575)	n.s.	2.54 (1.63)	3.13 (1.58)	n.s.	2.25 (1.83)	3.06 (1.66)
Norms	Others behavior (relatives)	0.97*	3.25 (1.138)	2.15 (1.137)	0.95*	3.73 (1.31)	2.45 (1.37)	0.81*	3.25 (1.49)	2.17 (1.15)
	Others behavior (neighbors)	1.55*	4.00 (1.044)	2.18 (1.287)	1.36*	4.15 (0.92)	2.59 (1.35)	2.03*	4.50 (0.53)	2.51 (1.28)
	Others approval	0.69*	2.67 (1.497)	1.71 (1.278)	0.64*	3.08 (1.55)	2.11 (1.47)	1.28*	3.63 (1.51)	1.80 (1.34)
	Personal obligation	1.29*	3.17 (1.467)	1.49 (1.119)	0.94*	2.81 (1.63)	1.47 (1.17)	0.87*	2.75 (1.98)	1.40 (0.96)
Ability factors	Confidence in performance	0.72*	2.58 (1.505)	1.61 (1.164)	1.79*	4.00 (1.44)	1.66 (1.16)	0.74*	2.38 (1.69)	1.40 (0.81)
	Confidence in performance (hurry)	0.78*	2.75 (1.765)	1.60 (1.130)	1.54*	3.77 (1.42)	1.72 (1.23)	1.56*	3.75 (1.75)	1.53 (0.97)
Self-regulation factors	Attention	0.8*	2.08 (1.505)	1.17 (0.558)	1.58*	3.23 (1.58)	1.27 (0.77)	1.89*	3.50 (1.60)	1.20 (0.61)
	Importance	1.45*	3.67 (1.557)	1.69 (1.148)	1.57*	3.77 (1.66)	1.59 (1.06)	1.43*	3.25 (1.67)	1.39 (0.77)
	Commitment	1.17*	3.17 (1.337)	1.70 (1.167)	1.72*	3.73 (1.48)	1.55 (1.01)	1.61*	3.50 (1.85)	1.29 (0.61)

n.s. = not significant.

*N* = 529; contact with river water: doers *n* = 47 and non-doers *n* = 482.

* *P* ≤0.001.

#### Contact with drain water.

In terms of the perceived risk of being exposed to AMR bacteria through contact with drain water, the “doers” were those who perceived no risk of AMR infection when they were in contact with drain water contained in the drains located within the household or elsewhere in the community. However, the “non-doers” were those who perceived a risk of AMR infection when they were in contact with drain water from the drains located within the household or elsewhere in the community. The study established that norms, such as others’ behavior (relatives) and others’ behavior (neighbors), and ability factors, such as confidence in performance (hurry), differed significantly between “doers” and “non-doers” in all the study settings (Table 3). This implied that the “doers” perceived that their relatives and neighbors were more frequently in contact with drain water compared with the “non-doers.” Further, the “doers” felt that there were no repercussions of being in contact with drain water than their counterparts. However, some behavioral factors differed among the three study locations. For instance, a medium significant effect was found in the norm factor “others’ approval” between the “doers” and “non-doers” in peri-urban (*d* = 0.55) and urban (*d* = 0.5) settings, implying that the doers were not discouraged from being in contact with drain water by their local leaders. Significant differences with medium to high Cohen’s *d* values were found in personal obligation and confidence in performance between the “doers” and “non-doers” in rural and peri-urban settings (Table 3). This means that these factors should be key targets for behavior change among doers of contact with drain water. A comparison between male and female study participants did not show great variation in the psychosocial factors influencing contact with drain water (Supplemental Tables 3 and 4).

**Table 3:** Contact with drain water: Doer and non-doer risk, attitude, norms, abilities, and self-regulation psychosocial factor means compared with analysis of variance

Factor Group	Behavioral Factors	Contact with Drain Water								
		Rural Setting			Peri-Urban Setting			Urban Setting		
		Cohen’s *d*	Doer’s, *M* (SD)	Non-Doer’s, *M* (SD)	Cohen’s *d*	Doer’s, *M* (SD)	Non-Doer’s, *M* (SD)	Cohen’s *d*	Doer’s, *M* (SD)	Non-Doer’s, *M* (SD)
Risk factors	Vulnerability	n.s.	4.29 (1.04)	4.33 (1.09)	n.s.	4.58 (0.96)	4.18 (1.17)	n.s.	4.71 (0.64)	4.39 (1.02)
Attitude factors	Pleasant	n.s.	1.42 (0.76)	1.25 (0.59)	n.s.	1.21 (0.92)	1.14 (0.51)	n.s.	1.19 (0.40)	1.07 (0.34)
	Time-consuming	–	–	–	–	–	–	–	–	–
	Disgusted	n.s.	4.03 (1.45)	4.10 (1.26)	n.s.	3.74 (1.82)	4.10 (1.37)	n.s.	4.48 (1.33)	4.39 (1.09)
	Care	n.s.	2.32 (1.60)	2.42 (1.49)	n.s.	2.26 (1.82)	2.65 (1.67)	n.s.	2.86 (1.96)	2.90 (1.71)
Norms	Others behavior (relatives)	0.81*	3.55 (0.99)	2.67 (1.17)	1.15*	4.16 (0.76)	2.99 (1.21)	0.66*	3.38 (0.92)	2.67 (1.22)
	Others behavior (neighbors)	0.6*	3.68 (0.91)	3.05 (1.17)	0.52*	3.68 (1.16)	3.06 (1.24)	0.62*	3.76 (1.04)	3.09 (1.12)
	Others approval	n.s.	1.81 (1.38)	1.67 (1.14)	0.55*	2.42 (1.87)	1.57 (1.11)	0.5*	1.81 (1.44)	1.27 (0.74)
	Personal obligation	0.59*	2.61 (1.75)	1.69 (1.32)	1.24*	3.63 (1.80)	1.67 (1.34)	n.s.	1.90 (1.58)	1.44 (1.14)
Ability factors	Confidence in performance	0.90*	3.42 (1.57)	2.12 (1.30)	1.06*	3.74 (1.59)	2.17 (1.35)	n.s.	2.24 (1.51)	1.69 (1.18)
	Confidence in performance (hurry)	0.68*	3.55 (1.59)	2.53 (1.41)	0.70*	3.74 (1.56)	2.69 (1.45)	0.92*	3.62 (1.63)	2.25 (1.32)
Self-regulation factors	Attention	n.s.	3.26 (1.44)	3.67 (1.40)	n.s.	3.84 (1.34)	4.12 (1.23)	n.s.	4.05 (1.24)	4.18 (1.09)
	Importance	n.s.	1.74 (1.12)	1.31 (0.79)	n.s.	1.26 (0.93)	1.24 (0.77)	n.s.	1.05 (0.22)	1.09 (0.36)
	Commitment	n.s.	3.58 (1.63)	3.76 (1.53)	n.s.	4.58 (1.02)	4.14 (1.37)	n.s.	3.95 (1.50)	4.26 (1.32)

n.s. = not significant.

*N* = 529; contact with Drain water: doers *n* = 73 and non-doers *n* = 456.

* *P* ≤0.001.

#### Contact with animal feces.

In terms of the perceived risk of being exposed to AMR bacteria through contact with animal feces, the “doers” were the study participants who perceived no risk of AMR infection when they were in contact with animal feces. In contrast, the “non-doers” were those who perceived a risk of AMR infection when they were in contact with animal feces. Overall ability and self-regulation factors appeared to be the most significant in terms of behaviors around contact with animal feces in relation to AMR bacteria exposure.

There was a significant difference between the “doers” and “non-doers” in relation to confidence in performance (hurry) across all three study settings (Table 4). This implies that the “doers” did not try to reduce contact with animal feces whenever they were in a hurry or busy. For the peri-urban and urban settings, significant differences with medium to high Cohen’s *d* values were also found in attention, importance, and commitment to controlling contact with animal feces. Further, a significant difference between the “doers” and “non-doers” was noted in others’ approval among study participants in the urban setting, and another difference between the two groups was found in personal obligation and confidence in performance in the peri-urban setting (Table 4). In the rural setting, a significant difference was noted between the “doers” and “non-doers” in the factor of others’ behavior (relative), implying that the “doers” perceived that their relatives were mostly in contact with animal feces. The study results did not show significant differences between male and female study participants in the psychosocial determinants related to contact with animal feces and exposure to AMR bacteria (Supplemental Tables 5 and 6).

**Table 4:** Contact with animal feces: Doer and non-doer risk, attitude, norms, abilities, and self-regulation psychosocial factor means compared with analysis of variance

Factors Group	Behavioral Factors	Contact with Animal Feces								
		Rural Setting			Peri-Urban Setting			Urban Setting		
		Cohen’s *d*	Doer’s, *M* (SD)	Non-Doer’s, *M* (SD)	Cohen’s *d*	Doer’s, *M* (SD)	Non-Doer’s, *M* (SD)	Cohen’s *d*	Doer’s, *M* (SD)	Non-Doer’s, *M* (SD)
Risk factors	Vulnerability	n.s.	4.22 (1.17)	4.09 (1.15)	n.s.	4.19 (1.21)	4.15 (1.13)	n.s.	4.54 (0.95)	4.12 (1.27)
Attitude factors	Pleasant	n.s.	1.59 (1.07)	1.45 (0.82)	n.s.	1.64 (1.13)	1.34 (0.91)	n.s.	1.35 (0.89)	1.16 (0.65)
	Time-consuming	–	–	–	–	–	–	–	–	–
	Disgusted	n.s.	3.31 (1.73)	3.32 (1.47)	n.s.	3.09 (1.75)	3.84 (1.51)	n.s.	3.58 (1.77)	4.13 (1.36)
	Care	n.s.	3.34 (1.71)	3.10 (1.58)	n.s.	3.45 (1.52)	3.54 (1.60)	n.s.	3.50 (1.66)	3.59 (1.59)
Norms	Others behavior (relatives)	0.91*	3.40 (1.13)	2.11 (1.07)	n.s.	2.87 (1.24)	3.06 (1.17)	n.s.	2.81 (1.10)	3.15 (1.25)
	Others behavior (neighbors)	n.s.	2.84 (1.06)	2.71 (1.01)	n.s.	2.89 (1.15)	3.08 (1.06)	n.s.	3.12 (0.99)	3.18 (1.13)
	Others approval	n.s.	2.50 (1.58)	2.01 (1.42)	n.s.	2.19 (1.56)	1.77 (1.30)	0.56*	2.27 (1.76)	1.45 (1.09)
	Personal obligation	n.s.	2.64 (1.64)	2.91 (1.62)	0.70*	2.62 (1.66)	3.72 (1.48)	n.s.	3.46 (1.73)	4.01 (1.37)
Ability factors	Confidence in performance	n.s.	3.02 (1.62)	3.43 (1.51)	0.69*	3.40 (1.61)	4.37 (1.15)	n.s.	3.81 (1.58)	4.15 (1.27)
	Confidence in performance (hurry)	0.7*	2.40 (1.28)	1.61 (0.96)	0.88*	2.15 (1.27)	1.26 (0.64)	1.13*	2.08 (1.16)	1.10 (0.38)
Self-regulation factors	Attention	n.s.	2.35 (1.58)	2.08 (1.39)	0.69*	2.13 (1.56)	4.37 (1.15)	0.72*	1.96 (1.37)	1.20 (0.64)
	Importance	n.s.	2.99 (1.59)	2.29 (1.35)	0.88*	2.55 (1.49)	1.26 (0.64)	0.87*	2.65 (1.67)	1.44 (1.06)
	Commitment	n.s.	2.69 (1.49)	2.39 (1.47)	0.69*	2.11 (1.45)	4.37 (1.15)	0.65*	2.73 (1.69)	1.80 (1.15)

n.s. = not significant.

*N* = 529; contact with animal feces: doers *n* = 159 and non-doers *n* = 370.

* *P* ≤0.001.

## DISCUSSION

The reduction of disease transmission from an unsafe environment is associated with good infrastructure.^27^ However, improving the current environmental infrastructure may take time, particularly in urban and peri-urban areas that have seen rapid population expansion.^28^ Therefore, in combination with these improvements, behavioral change techniques that target risky behaviors should be prioritized around these high-risk environments. Such behavior change interventions may not only benefit AMR infection reduction but also provide further social good in the reduction of other communicable diseases, including typhoid^29^ and diarrheal disease (i.e., cholera).

To support the development of effective behavior change interventions, this study sought to understand individual perceptions of risk of AMR infection through environmental exposure and to further assess the psychosocial factors related to high-risk behaviors that may be contributing to AMR infection transmission through these contacts with open water sources, drains, and both animal and human feces.

For many, the perceived risk of AMR infection is still commonly associated with antimicrobial stewardship and hospital-acquired infections,^5^ and there is little understanding of the role the wider environment plays in the proliferation and transmission of these infections. This was validated because more than half of the study participants were aware of AMR bacteria, but this was primarily linked with knowledge of someone who had experienced an AMR infection, which could be attributed to the available information about the causation of the AMR-related diagnosis. This is not surprising given that AMR infection messaging mostly focuses on the role of private traders and self-medication in AMR infection transmission^30^ and is not associated with environmental factors. Females also indicated a higher level of awareness than their male counterparts of the presence of risks of AMR infection. Because women have more positive health-seeking behavior^31^^–^^33^ and mostly accompany their relatives, including children, to the hospital when sick,^34^^,^^35^ they are exposed to more health messages than their male counterparts, which may have increased their AMR infection risk perception. Relatedly, respondents indicated that the transmission of these infections could result from exposure to human and animal feces, which may be related to previous awareness of fecal–oral route disease transmission.^36^ Nevertheless, high-risk environmental pathways for AMR bacteria exposure were identified from observations and sampling at the household and wider community levels in all three settings^7^ and elsewhere.^37^^,^^38^

Thus, it could be assumed that current AMR infection knowledge is associated with health facility visitation and previous health education sessions that focused on interrupting fecal–oral route transmission. With this in mind, AMR infection awareness campaigns should widen their focus to include the wider drivers and sources of AMR infection and target all study locations equally because individual AMR risk perception was low. There is also a need to use multiple communication channels that apply various touch points to maximize male participation in AMR infection risk awareness campaigns.

Despite the sociodemographic differences, all study locations were influenced by normative and ability factors in making decisions about the use of river and drain water for domestic activities. Further, self-regulation (attention, importance, and commitment) and attitude factors (pleasant) were also strong predictors for the use of river water behavior. Thus, information about others’ behavior, guided practice, and model behavior should be considered in a behavior change intervention for improved practices on the use of river water, contact with drain water, and contact with animal feces in all the study areas. Previous studies have proven that influence from others plays an important role in one’s behavior about water and sanitation.^39^ Hence, there is a call for corresponding behavior change techniques (BCTs) to elicit behavior change.^40^ Thus, community group meetings could be considered because they have proven to strengthen community social norms.^41^ Such group meetings would involve role models to reinforce positive group identity. Availability and access to safe water points for households increase the confidence to use safe water for domestic purposes.^14^ This confirms the need to intensify the accessibility and affordability of safe water at the household level. Role models should be incorporated to demonstrate to others how they sustain using safe water in their homes.

Studies in Ethiopia and Zimbabwe found that self-regulation (remembering) and ability (infrastructure) factors were strong predictors of the performance of handwashing behavior.^13^^,^^42^ In addition, self-regulation (remembering and commitment), risk perception (perceived risk and benefits), and norms (others behavior) were associated with behaviors pertaining to safe water household usage in Bangladesh and Chad.^12^^,^^43^ For sanitation and food hygiene behaviors, attitude (expensive and like), norms (others’ behaviors), self-regulation (remembering and planning), and risk perception were identified as behavioral determinants in Malawi, Ghana, and Burundi.^14^^,^^44^^–^^46^ In these studies, the following BCTs were identified from the RANAS model of behavior change BCT catalogue^11^ for intervention delivery: information about and assessment of personal risk, infrastructure promotion, memory aids, guided behavioral and practice, norm identification, and behavior modeling and demonstration. Importantly, behavioral determinants identified in these previous studies have also been observed in this study influencing the use of river water, contact with drain water, and contact with animal feces. This implies that AMR infection awareness promotion should be included in wider WASH programs and could be used as a leverage point to gain much-needed rapid momentum about AMR infection awareness.

### Implication for practice.

In this study, the norm factors (i.e., others’ behavior and others’ approval) have been found to influence the practice pertaining to the use of river and drain water for domestic activities. Thus, corresponding BCTs for normative factors should be taken into consideration, such as community meetings, household visits, the use of role models, and public pledges, all of which have been proven to strengthen behavioral normative attributes.^13^^,^^47^ Similarly, ability factors (confidence in performance) have been found to influence the three targeted behaviors; thus, BCTs pertaining to ability factors should be considered in an intervention.

Corresponding BCTs related to attitude factors, such as describing feelings about the performance and consequences of the behavior, should be considered for use in guiding river water behavior in all the study areas. In addition, information about personal AMR infection risk-related activities should be included in an intervention aimed at the use of river water in the urban setting. Such information should include practical demonstrations illustrating AMR infection transmission pathways through river and drain water to reinforce the vulnerability factors. For contact with animal feces, self-regulation factors (attention, importance, and commitment) were strong predictors in peri-urban and rural settings, whereas the ability factor (confidence in performance) was significant in all the study areas. As such, BCTs related to the identified self-regulation factors should be considered in an intervention. For instance, public commitment should be included for household members and local leaders because they have been proven to influence the uptake of positive behaviors.^39^

### Study limitations.

This study had lower male participation than had been planned. Future study designs should consider following men in their respective workplaces or places of business because they are often not found in the household. Self-reported data are liable to bias.^48^ Nevertheless, observations were conducted on a number of variables (including access to safe water and the presence of animal feces at the household level) that have been reported elsewhere.^7^ The coronavirus disease 2019 pandemic likely impacted our results because data collection was delayed for ∼5 months, which resulted in collecting data for a shorter period than initially planned because the project life span had finished.

## CONCLUSION

The findings of this study indicate that slightly more than half of the study participants were aware of AMR infection. However, few participants perceived themselves as being at risk of AMR infection through river and drain water exposure. Importantly, limited knowledge of the roles of fecal matter and river and drain water in AMR infection transmission acts as a call for more effort to be expended on AMR infection risk exposure awareness. Such awareness campaigns should target all the study locations and use various touch points to maximize the participation of both women and men; they should also be incorporated into wider WASH promotion campaigns. Selected psychosocial factors, including attitudinal, normative, ability, and self-regulation factors, have been isolated as strong predictors for the success of an AMR infection behavior change intervention that focuses on the environmental factors of interest: the use of river and drain water and being in contact with animal feces. Thus, although improving sanitary facilities and drainage systems in the study locations will be of significant value, context-specific behavior change initiatives can be an effective tool in these economically challenged settings in the short to medium term.

## Supplemental Materials

10.4269/ajtmh.24-0253 Supplemental Materials

## References

[1] Maillard J-Y Bloomfield SF Courvalin P Essack SY Gandra S Gerba CP Rubino JR Scott EA, 2020. Reducing antibiotic prescribing and addressing the global problem of antibiotic resistance by targeted hygiene in the home and everyday life settings: A position paper. *Am J Infect Control* 48: 1090–1099. 32311380 10.1016/j.ajic.2020.04.011 PMC7165117

[2] Woolhouse M Farrar J, 2014. Policy: An intergovernmental panel on antimicrobial resistance. Nature 509: 555–557. 24877180 10.1038/509555a

[3] Murray CJL , 2022. Global burden of bacterial antimicrobial resistance in 2019: A systematic analysis. Lancet 399: 629–655. 35065702 10.1016/S0140-6736(21)02724-0 PMC8841637

[4] Chandler CIR Nayiga S, 2023. Antimicrobial resistance in cities: An overlooked challenge that requires a multidisciplinary approach. Lancet 401: 627–629. 36403585 10.1016/S0140-6736(22)02351-0

[5] Fuhrmeister ER , 2023. Evaluating the relationship between community water and sanitation access and the global burden of antibiotic resistance: An ecological study. *Lancet Microbe* 4: e591–e600. 37399829 10.1016/S2666-5247(23)00137-4 PMC10393780

[6] World Health Organization, 2016. *Global Action Plan on Antimicrobial Resistance*. Available at: https://www.who.int/publications-detail-redirect/9789241509763. Accessed December 9, 2016.

[7] Cocker D , 2023. Investigating One Health risks for human colonisation with extended spectrum β-lactamase-producing *Escherichia coli* and *Klebsiella pneumoniae* in Malawian households: A longitudinal cohort study. Lancet Microbe 4: e534–e543. 37207684 10.1016/S2666-5247(23)00062-9 PMC10319635

[8] Sammarro M , 2023. Risk factors, temporal dependence, and seasonality of human extended-spectrum β-lactamases-producing *Escherichia coli* and *Klebsiella pneumoniae* Colonization in Malawi: A longitudinal model-based approach. *Clin Infect Dis* 77: 1–8. 36869813 10.1093/cid/ciad117 PMC10320086

[9] Amato HK , 2023. Risk factors for extended-spectrum beta-lactamase (ESBL)-producing *E. coli* carriage among children in a food animal-producing region of Ecuador: A repeated measures observational study. PLoS Med 20: e1004299. 37831716 10.1371/journal.pmed.1004299 PMC10621961

[10] Wang Y , 2022. Quantitative assessment of exposure to fecal contamination in urban environment across nine cities in low-income and lower-middle-income countries and a city in the United States. *Sci Total Environ* 806: 151273. 34718001 10.1016/j.scitotenv.2021.151273 PMC8651627

[11] MoslerContzen N, 2016. Systematic Behavior Change in Water, Sanitation and Hygiene. A Practical Guide Using the RANAS Approach. Dubendorf, Switzerland: Eawag.

[12] Lilje J Mosler H-J, 2018. Effects of a behavior change campaign on household drinking water disinfection in the Lake Chad basin using the RANAS approach. Sci Total Environ 619-620: 1599–1607. 29111247 10.1016/j.scitotenv.2017.10.142

[13] Friedrich MND Binkert ME Mosler HJ, 2017. Contextual and psychosocial determinants of effective handwashing technique: Recommendations for interventions from a case study in Harare, Zimbabwe. *Am J Trop Med Hyg* 96: 430–436. 28044046 10.4269/ajtmh.16-0553 PMC5303049

[14] Chidziwisano K Slekiene J Kumwenda S Mosler H-J Morse T, 2019. Toward complementary food hygiene practices among child caregivers in rural Malawi. *Am J Trop Med Hyg* 101: 294–303. 31237230 10.4269/ajtmh.18-0639 PMC6685574

[15] Cocker D , 2022. Drivers of resistance in Uganda and Malawi (DRUM): A protocol for the evaluation of One-Health drivers of extended spectrum beta lactamase (ESBL) resistance in low-middle income countries (LMICs). Wellcome Open Res 7: 55. 38817338 10.12688/wellcomeopenres.17581.2 PMC11137479

[16] Darton TC ; STRATAA Study Consortium, 2017. The STRATAA study protocol: A programme to assess the burden of enteric fever in Bangladesh, Malawi and Nepal using prospective population census, passive surveillance, serological studies and healthcare utilisation surveys. BMJ Open 7: e016283. 10.1136/bmjopen-2017-016283 PMC5726077 28674145

[17] Kazembe LN Mathanga DP, 2016. Estimating risk factors of urban malaria in Blantyre, Malawi: A spatial regression analysis. Asian Pac J Trop Biomed 6: 376–381.

[18] Kalina M Kwangulero J Ali F Abera YG Tilley E, 2022. “Where does it go?”: Perceptions and problems of riverine and marine litter amongst South Africa and Malawi’s urban poor. PLOS Water 1: e0000013.

[19] Ewing VL Tolhurst R Kapinda A Richards E Terlouw DJ Lalloo DG, 2016. Increasing understanding of the relationship between geographic access and gendered decision-making power for treatment-seeking for febrile children in the Chikwawa district of Malawi. Malar J 15: 521. 27776549 10.1186/s12936-016-1559-0 PMC5078939

[20] Nightingale R Lesosky M Flitz G Rylance SJ Meghji J Burney P Balmes J Mortimer K, 2019. Noncommunicable respiratory disease and air pollution exposure in Malawi (CAPS). A cross-sectional study. Am J Respir Crit Care Med 199: 613–621. 30141966 10.1164/rccm.201805-0936OC PMC6396863

[21] Government of Malawi, 2016. Malawi Demographic and Health Survey 2015–16. Zomba, Malawi: National Statistical Office.

[22] Ajzen I, 1991. The theory of planned behavior. Organ Behav Hum Decis Process 50: 179–211.

[23] Bandura A, 1977. Self-efficacy: Toward a unifying theory of behavioral change. *Psychol Rev* 84: 191–215. 847061 10.1037//0033-295x.84.2.191

[24] Cialdini RB Trost MR, 1998. Social influence: Social norms, conformity and compliance. Gilbert DT, Fiske ST, Lindzey G, eds. The Handbook of Social Psychology Vols 1-2, 4th ed. New York, NY: McGraw-Hill, 151–192.

[25] Schwarzer R, 2008. Modeling health behavior change: How to predict and modify the adoption and maintenance of health behaviors. Appl Psychol 57: 1–29.

[26] Lakshminarasimhappa MC, 2022. Web-based and smart mobile app for data collection: Kobo Toolbox/Kobo Collect. J Indian Library Assoc 57: 72–79.

[27] Freeman MC , 2013. Integration of water, sanitation, and hygiene for the prevention and control of neglected tropical diseases: A rationale for inter-sectoral collaboration. *PLoS Negl Trop Dis* 7: e2439. 24086781 10.1371/journal.pntd.0002439 PMC3784463

[28] UNICEF, 2019. Global Framework for Urban Water, Sanitation and Hygiene. Geneva, Switzerland: UNICEF.

[29] Gauld JS , 2020. Domestic river water use and risk of typhoid fever: Results from a case-control study in Blantyre, Malawi. *Clin Infect Dis* 70: 1278–1284. 31144715 10.1093/cid/ciz405

[30] Lusti-Narasimhan M Pessoa-Silva CL Temmerman M, 2013. Moving forward in tackling antimicrobial resistance: WHO actions. Sex Transm Infect 89 Suppl 4: iv57–iv59. 24202206 10.1136/sextrans-2012-050910

[31] Hjelm K Atwine F, 2011. Health-care seeking behaviour among persons with diabetes in Uganda: An interview study. *BMC Int Health Hum Rights* 11: 11. 21943099 10.1186/1472-698X-11-11 PMC3213135

[32] Galdas PM Cheater F Marshall P, 2005. Men and health help-seeking behaviour: Literature review. *J Adv Nurs* 49: 616–623. 15737222 10.1111/j.1365-2648.2004.03331.x

[33] Abdulraheem IS, 2007. Health needs assessment and determinants of health-seeking behaviour among elderly Nigerians: A house-hold survey. *Ann Afr Med* 6: 58–63. 18240704 10.4103/1596-3519.55715

[34] Hou X Ma N, 2013. The effect of women’s decision-making power on maternal health services uptake: Evidence from Pakistan. *Health Policy Plan* 28: 176–184. 22522771 10.1093/heapol/czs042

[35] Ganle JK Dery I, 2015. What men don’t know can hurt women’s health’: A qualitative study of the barriers to and opportunities for men’s involvement in maternal healthcare in Ghana. *Reprod Health* 12: 93. 26452546 10.1186/s12978-015-0083-y PMC4600282

[36] Watson JA Ensink JHJ Ramos M Benelli P Holdsworth E Dreibelbis R Cumming O, 2017. Does targeting children with hygiene promotion messages work? The effect of handwashing promotion targeted at children, on diarrhoea, soil-transmitted helminth infections and behaviour change, in low- and middle-income countries. *Trop Med Int Health* 22: 526–538. 28244191 10.1111/tmi.12861

[37] Amin N , 2019. Quantitative assessment of fecal contamination in multiple environmental sample types in urban communities in Dhaka, Bangladesh using SaniPath microbial approach. Plos One 14: e0221193. 31841549 10.1371/journal.pone.0221193 PMC6913925

[38] Mwapasa T Chidziwisano K Mphasa M Cocker D Rimella L Amos S Feasey N Morse T, 2024. Key environmental exposure pathways to antimicrobial resistant bacteria in southern Malawi: A SaniPath approach. *Sci Total Environ* 945: 174142. 38906299 10.1016/j.scitotenv.2024.174142 PMC11234251

[39] Nelson S Drabarek D Jenkins A Negin J Abimbola S, 2021. How community participation in water and sanitation interventions impacts human health, WASH infrastructure and service longevity in low-income and middle-income countries: A realist review. BMJ Open 11: e053320. 10.1136/bmjopen-2021-053320 PMC8640661 34857572

[40] Mosler H-J, 2012. A systematic approach to behavior change interventions for the water and sanitation sector in developing countries: A conceptual model, a review, and a guideline. *Int J Environ Health Res* 22: 431–449. 22292899 10.1080/09603123.2011.650156

[41] Waterkeyn J Cairncross S, 2005. Creating demand for sanitation and hygiene through Community Health Clubs: A cost-effective intervention in two districts in Zimbabwe. Soc Sci Med 61: 1958–1970. 15927329 10.1016/j.socscimed.2005.04.012

[42] Contzen N Jennifer I, 2015. Social-cognitive factors mediating intervention effects on handwashing: A longitudinal study. J Behav Med 38: 956–969. 26243641 10.1007/s10865-015-9661-2

[43] Inauen J Mosler H-J, 2016. Mechanisms of behavioural maintenance: Long-term effects of theory-based interventions to promote safe water consumption. *Psychol Health* 31: 166–183. 26304476 10.1080/08870446.2015.1085985

[44] Harter M Inauen J Mosler H-J, 2020. How does community-led total sanitation (CLTS) promote latrine construction, and can it be improved? A cluster-randomized controlled trial in Ghana. Soc Sci Med 245: 112705. 31838334 10.1016/j.socscimed.2019.112705 PMC6983942

[45] Seimetz E Slekiene J Friedrich MND Mosler H-J, 2017. Identifying behavioural determinants for interventions to increase handwashing practices among primary school children in rural Burundi and urban Zimbabwe. *BMC Res Notes* 10: 280. 28705260 10.1186/s13104-017-2599-4 PMC5513052

[46] Chidziwisano K Slekiene J Mosler H-J Morse T, 2020. Improving complementary food hygiene behaviors using the risk, attitude, norms, ability, and self-regulation approach in rural Malawi. *Am J Trop Med Hyg* 102: 1104–1115. 32100679 10.4269/ajtmh.19-0528 PMC7204602

[47] Colbourn T , 2015. Cost-effectiveness and affordability of community mobilisation through women’s groups and quality improvement in health facilities (MaiKhanda trial) in Malawi. *Cost Eff Resour Alloc* 13: 1. 25649323 10.1186/s12962-014-0028-2 PMC4299571

[48] Curtis V Cousens S Mertens T Traore E Kanki B Diallo I, 1993. Structured observations of hygiene behaviours in Burkina Faso: Validity, variability, and utility. Bull World Health Organ 71: 23–32. 8440034 PMC2393438

