# Supplementary material for: Risk Perception and Psychosocial Factors Influencing Exposure to Antimicrobial Resistance through Environmental Pathways in Malawi
Source: Am J Trop Med Hyg. 2024 Dec 3;112(2):355–63. doi: 10.4269/ajtmh.24-0253 (PMC11803648; doi:10.4269/ajtmh.24-0253)
Supplement: Supplemental Materials [file tpmd240253.SD1.pdf]

Risk perception and psychosocial factors influencing exposure to antimicrobial resistance through environmental pathways in Malawi

Supplementary material

S1: Use of river water factors by gender – female

Descriptives									
		N	Mean	Std. Deviation	Std. Error	95% Confidence Interval for Mean		Minimum	Cohen's D test
						Lower Bound	Upper Bound		
46.How pleasant is it to use river water for household purposes?	0	299	1.68	1.18	0.07	1.55	1.82	1	0.93
	1	26	3.08	1.76	0.35	2.36	3.79	1	
	Total	325	1.79	1.29	0.07	1.65	1.93	1	
47.How time consuming is it to use river water for household purposes	0	299	3.29	1.64	0.09	3.10	3.48	1	0.72
	1	26	2.15	1.52	0.30	1.54	2.77	1	
	Total	325	3.20	1.66	0.09	3.02	3.38	1	
48.Do you (or would you) feel ashamed using river water for household purposes?	0	299	2.00	1.56	0.09	1.82	2.17	1	0.29
	1	26	1.58	1.30	0.26	1.05	2.10	1	
	Total	325	1.96	1.54	0.09	1.80	2.13	1	
50.Do you feel disgusted using	0	299	3.36	1.75	0.10	3.17	3.56	1	

river water for household purposes?									
	1	26	2.42	1.47	0.29	1.83	3.02	1	0.58
	Total	325	3.29	1.75	0.10	3.10	3.48	1	
52.How convenient is it for you to use river water?	0	299	2.57	1.62	0.09	2.39	2.76	1	
	1	26	4.12	1.42	0.28	3.54	4.69	1	1.01
	Total	325	2.70	1.65	0.09	2.51	2.88	1	
53.Do you care if the water from the river is safe to use?	0	299	3.05	1.64	0.10	2.86	3.24	1	
	1	26	2.65	1.77	0.35	1.94	3.37	1	0.23
	Total	325	3.02	1.65	0.09	2.84	3.20	1	
54. What do you think, how many of your relatives use river water for household purposes?	0	299	2.27	1.22	0.07	2.13	2.41	1	
	1	26	3.58	1.30	0.26	3.05	4.10	1	1.04
	Total	325	2.37	1.28	0.07	2.23	2.51	1	
55.What do you think, how many of your neighbours use river water for household purposes?	0	299	2.46	1.33	0.08	2.31	2.61	1	
	1	26	4.19	0.94	0.18	3.81	4.57	2	1.50
	Total	325	2.60	1.38	0.08	2.45	2.75	1	

56.What do you think, how many leaders (religious, community etc) use river water for household purposes?	0	299	1.61	0.93	0.05	1.50	1.71	1	
	1	26	2.54	1.27	0.25	2.02	3.05	1	0.84
	Total	325	1.68	0.99	0.05	1.57	1.79	1	
57.People who are important in the community, like religious leaders, chiefs, block leaders, councillors, etc; how much do they approve that you should be using river water?	0	299	1.94	1.43	0.08	1.78	2.11	1	
	1	26	3.35	1.47	0.29	2.75	3.94	1	0.97
	Total	325	2.06	1.48	0.08	1.89	2.22	1	
58.Do you feel a personal obligation to use river water for household purpose?	0	299	1.46	1.10	0.06	1.34	1.59	1	
	1	26	2.77	1.48	0.29	2.17	3.37	1	1.00
	Total	325	1.57	1.19	0.07	1.44	1.70	1	
59.How confident are you that you can use river water for	0	299	1.56	1.05	0.06	1.44	1.67	1	

domestic purposes?									
	1	26	3.23	1.66	0.32	2.56	3.90	1	1.21
	Total	325	1.69	1.20	0.07	1.56	1.82	1	
60.Imagine that you are in a hurry How confident are you that you can use river water for household purposes?	0	299	1.64	1.20	0.07	1.50	1.78	1	
	1	26	3.38	1.65	0.32	2.72	4.05	1	1.21
	Total	325	1.78	1.33	0.07	1.63	1.92	1	
61.For the past 24 hours, how often did it happen that you wanted to use river water for domestic purposes but you could not do so because it was not available or you forgot	0	299	1.07	0.37	0.02	1.03	1.11	1	
	1	26	1.46	0.99	0.19	1.06	1.86	1	0.52
	Total	325	1.10	0.46	0.03	1.05	1.15	1	
62.How much do you pay attention that you always ensure that you have river water for domestic purposes?	0	299	1.21	0.69	0.04	1.14	1.29	1	
	1	26	3.35	1.62	0.32	2.69	4.00	1	1.71

	Total	325	1.38	0.99	0.05	1.28	1.49	1	
63. How important is it for you to use river water for domestic purposes?	0	299	1.58	1.04	0.06	1.46	1.69	1	
	1	26	4.00	1.50	0.29	3.40	4.60	1	1.88
	Total	325	1.77	1.27	0.07	1.63	1.91	1	
64.How committed are you using river water for domestic purposes?	0	299	1.53	1.01	0.06	1.41	1.64	1	
	1	26	3.69	1.52	0.30	3.08	4.31	1	1.68
	Total	325	1.70	1.21	0.07	1.57	1.83	1	
22.If you use river water in your household, how high is the risk that you get an infection which may be resistant to antibiotics?	0	299	4.71	0.71	0.04	4.63	4.79	1	
	1	26	4.46	0.81	0.16	4.13	4.79	2	0.33
	Total	325	4.69	0.72	0.04	4.61	4.77	1	

S2: Use of river water factors by gender – male

Descriptives									
		N	Mean	Std. Deviation	Std. Error	95% Confidence Interval for Mean		Minimum	Cohen's D test
						Lower Bound	Upper Bound		
46.How pleasant is it to use river water for household purposes?	0	183	1.49	0.89	0.07	1.36	1.62	1	
	1	18	2.83	1.62	0.38	2.03	3.64	1	1.03
	Total	201	1.61	1.04	0.07	1.47	1.76	1	
47.How time consuming is it to use river water for household purposes	0	183	3.62	1.55	0.11	3.39	3.84	1	
	1	18	1.83	1.34	0.32	1.17	2.50	1	1.23
	Total	201	3.46	1.61	0.11	3.23	3.68	1	
48.Do you (or would you) feel ashamed using river water for household purposes?	0	183	2.50	1.75	0.13	2.25	2.76	1	
	1	18	1.78	1.56	0.37	1.00	2.55	1	0.44
	Total	201	2.44	1.74	0.12	2.20	2.68	1	
50.Do you feel disgusted using river water for household purposes?	0	183	3.75	1.59	0.12	3.52	3.98	1	
	1	18	2.39	1.46	0.34	1.66	3.12	1	0.89
	Total	201	3.63	1.63	0.11	3.40	3.85	1	
52.How convenient is it for you to use river water?	0	183	2.48	1.68	0.12	2.23	2.72	1	
	1	18	4.22	1.22	0.29	3.62	4.83	1	1.19
	Total	201	2.63	1.72	0.12	2.39	2.87	1	
53.Do you care if the water from the river is safe to use?	0	183	2.95	1.56	0.12	2.72	3.17	1	
	1	18	2.50	1.65	0.39	1.68	3.32	1	0.28
	Total	201	2.91	1.57	0.11	2.69	3.12	1	
54. What do you think, how many of your relatives use river water for household purposes?	0	183	2.26	1.24	0.09	2.08	2.44	1	
	1	18	3.39	1.33	0.31	2.73	4.05	1	0.87

	Total	201	2.36	1.29	0.09	2.18	2.54	1	
55.What do you think, how many of your neighbours use river water for household purposes?	0	183	2.38	1.30	0.10	2.19	2.57	1	
	1	18	4.28	0.75	0.18	3.90	4.65	2	1.78
	Total	201	2.55	1.37	0.10	2.36	2.74	1	
56.What do you think, how many leaders (religious, community etc) use river water for household purposes?	0	183	1.84	1.11	0.08	1.67	2.00	1	
	1	18	3.00	1.37	0.32	2.32	3.68	1	0.93
	Total	201	1.94	1.18	0.08	1.78	2.10	1	
57.People who are important in the community, like religious leaders, chiefs, block leaders, councillors, etc; how much do they approve that you should be using river water?	0	183	1.73	1.25	0.09	1.55	1.91	1	
	1	18	2.67	1.64	0.39	1.85	3.48	1	0.64
	Total	201	1.81	1.31	0.09	1.63	1.99	1	
58.Do you feel a personal obligation to use river water for household purpose?	0	183	1.45	1.08	0.08	1.29	1.61	1	
	1	18	3.06	1.80	0.42	2.16	3.95	1	1.08
	Total	201	1.59	1.25	0.09	1.42	1.77	1	
59.How confident are you that you can use river water for domestic purposes?	0	183	1.55	1.08	0.08	1.39	1.71	1	
	1	18	3.39	1.72	0.41	2.53	4.24	1	1.28
	Total	201	1.72	1.26	0.09	1.54	1.89	1	
60.Imagine that you are in a hurry How confident are you that you can use river water for household purposes?	0	183	1.60	0.98	0.07	1.45	1.74	1	
	1	18	3.67	1.57	0.37	2.89	4.45	1	1.58
	Total	201	1.78	1.20	0.08	1.61	1.95	1	
61.For the past 24 hours, how often did it happen that you wanted to use river water for domestic purposes but you could not do so because it was not available or you forgot	0	183	1.07	0.33	0.02	1.02	1.12	1	
	1	18	1.72	1.41	0.33	1.02	2.42	1	0.64
	Total	201	1.13	0.55	0.04	1.05	1.21	1	

62.How much do you pay attention that you always ensure that you have river water for domestic purposes?	0	183	1.19	0.55	0.04	1.11	1.27	1	
	1	18	2.39	1.50	0.35	1.64	3.14	1	1.06
	Total	201	1.30	0.76	0.05	1.19	1.40	1	
63. How important is it for you to use river water for domestic purposes?	0	183	1.54	0.99	0.07	1.40	1.69	1	
	1	18	3.00	1.64	0.39	2.18	3.82	1	1.07
	Total	201	1.67	1.14	0.08	1.51	1.83	1	
64.How committed are you using river water for domestic purposes?	0	183	1.49	0.92	0.07	1.35	1.62	1	
	1	18	3.17	1.47	0.35	2.44	3.90	1	1.37
	Total	201	1.64	1.09	0.08	1.48	1.79	1	
22.If you use river water in your household, how high is the risk that you get an infection which may be resistant to antibiotics?	0	183	4.67	0.81	0.06	4.55	4.78	1	
	1	18	4.28	1.23	0.29	3.67	4.89	1	0.37
	Total	201	4.63	0.86	0.06	4.51	4.75	1	

S3: Contact with drain water by gender – male

Descriptives									
		N	Mean	Std. Deviation	Std. Error	95% Confidence Interval for Mean		Minimum	Cohen's D test
						Lower Bound	Upper Bound		
66.How pleasant is it to be in contact with drain water?	0	171	1.18	0.51	0.04	1.10	1.25	1	
	1	30	1.30	0.65	0.12	1.06	1.54	1	0.21
	Total	201	1.19	0.54	0.04	1.12	1.27	1	
_67_Do_you_feel_ashamed_being_i	0	171	2.89	1.74	0.13	2.63	3.16	1	
	1	30	2.90	1.94	0.35	2.18	3.62	1	0.00
	Total	201	2.90	1.76	0.12	2.65	3.14	1	
69.Do you feel disgusted being in contact with drain water?	0	171	4.29	1.14	0.09	4.11	4.46	1	
	1	30	4.20	1.35	0.25	3.70	4.70	1	0.07
	Total	201	4.27	1.17	0.08	4.11	4.44	1	
71.Do you care if the drain water you come in contact with is safe?	0	171	2.54	1.64	0.13	2.29	2.79	1	
	1	30	2.57	1.76	0.32	1.91	3.22	1	0.02
	Total	201	2.54	1.66	0.12	2.31	2.77	1	
72.What do you think, how many of your relatives are in contact with drain water?	0	171	2.98	1.25	0.10	2.79	3.17	1	
	1	30	3.90	0.92	0.17	3.56	4.24	2	0.83
	Total	201	3.12	1.25	0.09	2.95	3.29	1	
73.What do you think, how many of your neighbours are in contact with drain water?	0	171	3.26	1.17	0.09	3.09	3.44	1	
	1	30	3.93	0.94	0.17	3.58	4.29	2	0.63
	Total	201	3.36	1.16	0.08	3.20	3.52	1	
74.What do you think, how many leaders (religious, community etc) are in contact with drain water?	0	171	2.49	1.22	0.09	2.30	2.67	1	
	1	30	3.67	1.09	0.20	3.26	4.07	2	1.02

	Total	201	2.66	1.27	0.09	2.48	2.84	1	
75. People who are important in the community, like religious leaders, chiefs, block leaders, Councillors, etc; how much do they approve being in contact with drain water?	0	171	1.47	1.00	0.08	1.32	1.62	1	
	1	30	1.77	1.52	0.28	1.20	2.34	1	0.23
	Total	201	1.51	1.10	0.08	1.36	1.66	1	
76.Do you feel personal obligation being in contact with drain water?	0	171	1.72	1.38	0.11	1.51	1.93	1	
	1	30	2.90	1.86	0.34	2.20	3.60	1	0.72
	Total	201	1.90	1.52	0.11	1.68	2.11	1	
77.How confident are you that you can be in contact with drain water?	0	171	2.11	1.35	0.10	1.91	2.32	1	
	1	30	3.17	1.66	0.30	2.55	3.79	1	0.70
	Total	201	2.27	1.45	0.10	2.07	2.47	1	
78. Imagine that you're in a hurry, for example you are late for the market. How confident are you that you can in contact with drain water?	0	171	2.60	1.45	0.11	2.38	2.82	1	
	1	30	3.60	1.52	0.28	3.03	4.17	1	0.67
	Total	201	2.75	1.50	0.11	2.54	2.96	1	
79.How confident are you that you can be in contact with drain water even if the chances are very minimal (eg there is no rain in the area)?	0	171	1.53	0.98	0.08	1.38	1.68	1	
	1	30	1.70	0.99	0.18	1.33	2.07	1	0.70
	Total	201	1.56	0.98	0.07	1.42	1.69	1	
80.Imagine that you are being careful in your activities, how confident are you that you can avoid being in contact with drain water?	0	171	3.76	1.45	0.11	3.54	3.98	1	
	1	30	4.07	1.44	0.26	3.53	4.60	1	0.21
	Total	201	3.81	1.44	0.10	3.61	4.01	1	
81.How much do you pay attention to avoiding contact with drain water?	0	171	4.04	1.30	0.10	3.84	4.23	1	

	1	30	3.63	1.35	0.25	3.13	4.14	1	0.30
	Total	201	3.98	1.31	0.09	3.79	4.16	1	
82.How important is it for you to be in contact with drain water?	0	171	1.22	0.68	0.05	1.12	1.32	1	
	1	30	1.53	1.11	0.20	1.12	1.95	1	0.34
	Total	201	1.27	0.76	0.05	1.16	1.37	1	
83.How committed are you to avoid being in contact with drain water?	0	171	4.06	1.44	0.11	3.84	4.28	1	
	1	30	4.17	1.29	0.24	3.69	4.65	1	0.08
	Total	201	4.07	1.42	0.10	3.88	4.27	1	
24.If you get in contact with drain water, how high is the risk that you get an infection which may be resistant to antibiotics?	0	171	4.29	1.06	0.08	4.13	4.45	1	
	1	30	4.37	1.07	0.19	3.97	4.76	1	0.08
	Total	201	4.30	1.06	0.07	4.15	4.45	1	

S4: Contact with drain water by gender – female

Descriptives									
		N	Mean	Std. Deviation	Std. Error	95% Confidence Interval for Mean		Minimum	Cohen's D test
						Lower Bound	Upper Bound		
66.How pleasant is it to be in contact with drain water?	0	285	1.15	0.50	0.03	1.09	1.21	1	
	1	40	1.30	0.79	0.13	1.05	1.55	1	0.23
	Total	325	1.17	0.54	0.03	1.11	1.23	1	
_67_Do_you_feel_ashamed_being_i	0	285	2.69	1.70	0.10	2.50	2.89	1	
	1	40	2.45	1.80	0.28	1.88	3.02	1	0.14
	Total	325	2.66	1.71	0.10	2.48	2.85	1	
69.Do you feel disgusted being in contact with drain water?	0	285	4.12	1.33	0.08	3.96	4.27	1	
	1	40	4.05	1.65	0.26	3.52	4.58	1	0.04
	Total	325	4.11	1.37	0.08	3.96	4.26	1	
71.Do you care if the drain water you come in contact with is safe?	0	285	2.72	1.64	0.10	2.53	2.91	1	
	1	40	2.40	1.81	0.29	1.82	2.98	1	0.19
	Total	325	2.68	1.66	0.09	2.50	2.86	1	
72.What do you think, how many of your relatives are in contact with drain water?	0	285	2.68	1.17	0.07	2.55	2.82	1	
	1	40	3.50	0.96	0.15	3.19	3.81	2	0.76
	Total	325	2.78	1.18	0.07	2.66	2.91	1	
73.What do you think, how many of your neighbours are in contact with drain water?	0	285	2.97	1.17	0.07	2.83	3.11	1	
	1	40	3.58	1.01	0.16	3.25	3.90	1	0.55
	Total	325	3.05	1.17	0.06	2.92	3.17	1	
74.What do you think, how many leaders (religious, community etc) are in contact with drain water?	0	285	2.27	1.16	0.07	2.13	2.40	1	

	1	40	3.03	1.10	0.17	2.67	3.38	1	0.67
	Total	325	2.36	1.18	0.07	2.23	2.49	1	
75. People who are important in the community, like religious leaders, chiefs, block leaders, Councillors, etc; how much do they approve being in contact with drain water?	0	285	1.55	1.06	0.06	1.42	1.67	1	
	1	40	2.15	1.56	0.25	1.65	2.65	1	0.45
	Total	325	1.62	1.15	0.06	1.50	1.75	1	
76.Do you feel personal obligation being in contact with drain water?	0	285	1.54	1.21	0.07	1.40	1.68	1	
	1	40	2.53	1.80	0.28	1.95	3.10	1	0.65
	Total	325	1.66	1.33	0.07	1.51	1.80	1	
77.How confident are you that you can be in contact with drain water?	0	285	1.94	1.27	0.08	1.79	2.09	1	
	1	40	3.15	1.69	0.27	2.61	3.69	1	0.81
	Total	325	2.09	1.39	0.08	1.93	2.24	1	
78. Imagine that you're in a hurry, for example you are late for the market. How confident are you that you can in contact with drain water?	0	285	2.45	1.38	0.08	2.28	2.61	1	
	1	40	3.70	1.59	0.25	3.19	4.21	1	0.84
	Total	325	2.60	1.47	0.08	2.44	2.76	1	
79.How confident are you that you can be in contact with drain water even if the chances are very minimal (eg there is no rain in the area)?	0	285	1.61	1.04	0.06	1.49	1.74	1	
	1	40	2.40	1.48	0.23	1.93	2.87	1	0.61
	Total	325	1.71	1.13	0.06	1.59	1.83	1	
80.Imagine that you are being careful in your activities, how confident are you that you can avoid being in contact with drain water?	0	285	3.65	1.52	0.09	3.47	3.82	1	
	1	40	3.83	1.50	0.24	3.35	4.30	1	0.12
	Total	325	3.67	1.51	0.08	3.50	3.83	1	

81.How much do you pay attention to avoiding contact with drain water?	0	285	3.94	1.25	0.07	3.80	4.09	1	
	1	40	3.68	1.44	0.23	3.21	4.14	1	0.20
	Total	325	3.91	1.28	0.07	3.77	4.05	1	
82.How important is it for you to be in contact with drain water?	0	285	1.19	0.62	0.04	1.12	1.27	1	
	1	40	1.30	0.79	0.13	1.05	1.55	1	0.15
	Total	325	1.21	0.64	0.04	1.14	1.28	1	
83.How committed are you to avoid being in contact with drain water?	0	285	4.03	1.42	0.08	3.86	4.19	1	
	1	40	3.83	1.63	0.26	3.30	4.35	1	0.13
	Total	325	4.00	1.44	0.08	3.85	4.16	1	
24.If you get in contact with drain water, how high is the risk that you get an infection which may be resistant to antibiotics?	0	285	4.29	1.13	0.07	4.16	4.42	1	
	1	40	4.60	0.81	0.13	4.34	4.86	1	0.32
	Total	325	4.33	1.10	0.06	4.21	4.45	1	

S5: Contact with animal faeces by gender – male

Descriptives									
		N	Mean	Std. Deviation	Std. Error	95% Confidence Interval for Mean		Minimum	
						Lower Bound	Upper Bound		
88.How pleasant is it to come in contact with animal faeces?	0	143	1.27	0.74	0.06	1.14	1.39	1	
	1	58	1.59	1.01	0.13	1.32	1.85	1	0.36
	Total	201	1.36	0.84	0.06	1.24	1.47	1	
89.Do you feel ashamed being in contact with animal faeces?	0	143	2.82	1.74	0.15	2.53	3.11	1	
	1	58	2.41	1.73	0.23	1.96	2.87	1	0.23
	Total	201	2.70	1.74	0.12	2.46	2.94	1	
90.Do you feel disgusted being in contact with animal faeces?	0	143	3.83	1.42	0.12	3.60	4.07	1	
	1	58	3.24	1.81	0.24	2.77	3.72	1	0.36
	Total	201	3.66	1.56	0.11	3.44	3.88	1	
91.Do you care if the animal faeces you come in contact with are safe?	0	143	3.45	1.60	0.13	3.19	3.72	1	
	1	58	3.48	1.67	0.22	3.04	3.92	1	0.02
	Total	201	3.46	1.62	0.11	3.24	3.69	1	
92.What do you think, how many of your relatives avoid being in contact with animal faeces?	0	143	2.89	1.12	0.09	2.70	3.07	1	
	1	58	2.83	1.17	0.15	2.52	3.14	1	0.05
	Total	201	2.87	1.13	0.08	2.71	3.03	1	
93.What do you think, how many of your neighbours avoid being in contact with animal faeces?	0	143	2.88	1.06	0.09	2.71	3.06	1	
	1	58	2.81	1.15	0.15	2.51	3.11	1	0.06
	Total	201	2.86	1.08	0.08	2.71	3.01	1	

94.People who are important in the community, like religious leaders, chiefs, block leaders, councillors, etc; how much do they support to avoid being in contact with animal faeces?	0	143	2.03	1.53	0.13	1.77	2.28	1	
	1	58	1.78	1.39	0.18	1.41	2.14	1	0.02
	Total	201	1.96	1.49	0.11	1.75	2.16	1	
95.Do you feel a personal obligation being in contact with animal faeces?	0	143	1.89	1.46	0.12	1.65	2.13	1	
	1	58	2.40	1.65	0.22	1.96	2.83	1	0.33
	Total	201	2.03	1.53	0.11	1.82	2.25	1	
96.How confident are you that you can avoid being in contact with animal faeces?	0	143	3.73	1.43	0.12	3.50	3.97	1	
	1	58	2.95	1.79	0.24	2.48	3.42	1	0.48
	Total	201	3.51	1.58	0.11	3.29	3.73	1	
97.Imagine that you are being careful in your activities: How confident are you that you can avoid being in contact with animal faeces?	0	143	4.17	1.29	0.11	3.95	4.38	1	
	1	58	3.60	1.60	0.21	3.18	4.02	1	0.39
	Total	201	4.00	1.41	0.10	3.81	4.20	1	
98.For the past 24 hours, how often were you in contact with animal faeces	0	143	1.31	0.76	0.06	1.19	1.44	1	
	1	58	2.00	1.03	0.13	1.73	2.27	1	0.76
	Total	201	1.51	0.90	0.06	1.39	1.64	1	
99.How much do you agree with the following statement? Avoiding being in contact with animal faeces is something you do before you realize you are doing it.	0	143	3.28	1.64	0.14	3.01	3.55	1	
	1	58	3.21	1.71	0.23	2.76	3.66	1	0.04
	Total	201	3.26	1.66	0.12	3.03	3.49	1	
100.How important is it for you to be in contact with animal faeces?	0	143	1.60	1.16	0.10	1.41	1.79	1	
	1	58	2.10	1.52	0.20	1.70	2.50	1	0.37
	Total	201	1.75	1.29	0.09	1.57	1.93	1	

101.How committed are you being in contact with animal faeces?	0	143	1.87	1.30	0.11	1.66	2.09	1	
	1	58	2.53	1.61	0.21	2.11	2.96	1	0.45
	Total	201	2.06	1.43	0.10	1.87	2.26	1	
102.How often do you talk about being in contact with animal faeces with others (e.g friends)?	0	143	2.21	1.43	0.12	1.97	2.45	1	
	1	58	2.50	1.48	0.19	2.11	2.89	1	0.20
	Total	201	2.29	1.45	0.10	2.09	2.50	1	
25.If household water and utensils are in contact with animals/animal faeces, how high is the risk that you get an infection which may be resistant to antibiotics?	0	143	4.12	1.17	0.10	3.93	4.31	1	
	1	58	4.24	1.16	0.15	3.94	4.55	1	0.11
	Total	201	4.15	1.16	0.08	3.99	4.32	1	

S6: Contact with animal faeces by gender – female

Descriptives									
		N	Mean	Std. Deviation	Std. Error	95% Confidence Interval for Mean		Minimum	Cohen's D test
						Lower Bound	Upper Bound		
88.How pleasant is it to come in contact with animal faeces?	0	227	1.32	0.85	0.06	1.21	1.43	1	
	1	98	1.57	1.10	0.11	1.35	1.79	1	0.254254
	Total	325	1.40	0.94	0.05	1.29	1.50	1	
89.Do you feel ashamed being in contact with animal faeces?	0	227	2.36	1.66	0.11	2.14	2.58	1	
	1	98	2.08	1.60	0.16	1.76	2.40	1	0.171838
	Total	325	2.28	1.64	0.09	2.10	2.46	1	
90.Do you feel disgusted being in contact with animal faeces?	0	227	3.81	1.50	0.10	3.61	4.01	1	
	1	98	3.36	1.72	0.17	3.01	3.70	1	0.281305
	Total	325	3.67	1.58	0.09	3.50	3.85	1	
91.Do you care if the animal faeces you come in contact with are safe?	0	227	3.43	1.61	0.11	3.22	3.64	1	
	1	98	3.37	1.64	0.17	3.04	3.70	1	0.039618
	Total	325	3.41	1.62	0.09	3.24	3.59	1	
92.What do you think, how many of your relatives avoid being in contact with animal faeces?	0	227	3.11	1.22	0.08	2.95	3.27	1	
	1	98	2.73	1.16	0.12	2.50	2.97	1	0.315095
	Total	325	3.00	1.21	0.07	2.86	3.13	1	
93.What do you think, how many of your neighbours avoid being in contact with animal faeces?	0	227	3.12	1.10	0.07	2.98	3.27	1	
	1	98	2.93	1.04	0.10	2.72	3.14	1	0.181942
	Total	325	3.06	1.09	0.06	2.95	3.18	1	

94.People who are important in the community, like religious leaders, chiefs, block leaders, councillors, etc; how much do they support to avoid being in contact with animal faeces?	0	227	1.93	1.46	0.10	1.74	2.12	1	
	1	98	2.02	1.62	0.16	1.69	2.35	1	0.058768
	Total	325	1.96	1.51	0.08	1.79	2.12	1	
95.Do you feel a personal obligation being in contact with animal faeces?	0	227	1.63	1.15	0.08	1.48	1.78	1	
	1	98	2.33	1.56	0.16	2.01	2.64	1	0.508638
	Total	325	1.84	1.32	0.07	1.70	1.98	1	
96.How confident are you that you can avoid being in contact with animal faeces?	0	227	3.53	1.61	0.11	3.32	3.74	1	
	1	98	2.69	1.63	0.16	2.37	3.02	1	0.516002
	Total	325	3.28	1.66	0.09	3.10	3.46	1	
97.Imagine that you are being careful in your activities: How confident are you that you can avoid being in contact with animal faeces?	0	227	3.97	1.38	0.09	3.79	4.15	1	
	1	98	3.11	1.62	0.16	2.79	3.44	1	0.573065
	Total	325	3.71	1.51	0.08	3.55	3.88	1	
98.For the past 24 hours, how often were you in contact with animal faeces	0	227	1.29	0.66	0.04	1.20	1.38	1	
	1	98	2.46	1.36	0.14	2.19	2.73	1	1.095364
	Total	325	1.64	1.07	0.06	1.53	1.76	1	
99.How much do you agree with the following statement? Avoiding being in contact with animal faeces is something you do before you realize you are doing it.	0	227	3.36	1.68	0.11	3.14	3.58	1	
	1	98	3.00	1.63	0.16	2.67	3.33	1	0.215853
	Total	325	3.25	1.67	0.09	3.07	3.43	1	
100.How important is it for you to be in contact with animal faeces?	0	227	1.50	1.05	0.07	1.36	1.64	1	
	1	98	2.29	1.57	0.16	1.97	2.60	1	0.586043
	Total	325	1.74	1.28	0.07	1.60	1.88	1	

101.How committed are you being in contact with animal faeces?	0	227	1.75	1.24	0.08	1.59	1.91	1	
	1	98	2.96	1.56	0.16	2.65	3.27	1	0.858577
	Total	325	2.11	1.45	0.08	1.96	2.27	1	
102.How often do you talk about being in contact with animal faeces with others (e.g friends)?	0	227	1.98	1.34	0.09	1.80	2.15	1	
	1	98	2.58	1.56	0.16	2.27	2.89	1	0.415866
	Total	325	2.16	1.43	0.08	2.00	2.32	1	
25.If household water and utensils are in contact with animals/animal faeces, how high is the risk that you get an infection which may be resistant to antibiotics?	0	227	4.10	1.22	0.08	3.94	4.26	1	
	1	98	4.26	1.16	0.12	4.02	4.49	1	0.129335
	Total	325	4.15	1.20	0.07	4.02	4.28	1	
